# Dissonance reduction as emotion regulation: Attitude change is related to positive emotions in the induced compliance paradigm

**DOI:** 10.1371/journal.pone.0209012

**Published:** 2018-12-17

**Authors:** Sebastian Cancino-Montecinos, Fredrik Björklund, Torun Lindholm

**Affiliations:** 1 Department of Psychology, Division of Personality, Social, and Developmental Psychology, Stockholm University, Stockholm, Sweden; 2 Department of Psychology, Division of Personality and Social Psychology, Lund University, Lund, Sweden; University of Warsaw, POLAND

## Abstract

The aim of this study was to clarify how positive and negative emotions are related to the common attitude-change effect in cognitive dissonance research. Drawing on appraisal theories of emotion, and emotion-regulation research, we predicted that negative emotions would be inversely related to attitude change, whereas positive emotions would be positively related to attitude change in the induced compliance paradigm. In two studies, participants (N = 44; N = 106) wrote a counter-attitudinal essay under the perception of high choice, and were later asked to state their emotions in relation to writing this essay, as well as to state their attitude. Results confirmed the predictions, even when controlling for baseline emotions. These findings untangled a previously unresolved issue in dissonance research, which in turn shows how important emotion theories are for the understanding of cognitive dissonance processes.

## Introduction

The theory of cognitive dissonance has been studied for more than sixty years and is still one the most important theories in social psychology [1], [2]. In short, dissonance theory holds that people have an inner motivation to keep their cognitions coherent–especially when the cognitions are important. When an individual experiences inconsistency between related cognitions, psychological discomfort will follow. In order to regain a sense of equilibrium, and reduce discomfort, people tend to engage in dissonance-reduction strategies. For instance, in the *induced compliance paradigm*, participants are asked to write an essay that contradicts their previously stated attitudes. The common prediction is that people will change their attitude in order to regain a sense of consistency–especially if they perceive that the decision to write the contradictory essay was freely chosen. Other dissonance-reduction strategies involve downplaying the importance of the counter-attitudinal behavior [3], downplaying the strength of the initial attitude, or even denying responsibility for the behavior [4]. Although current dissonance research has contributed many insightful findings concerning attitudinal changes associated with dissonance, as yet there has been little focus on the experience of dissonance per se, particularly on the emotional aspects of dissonance. This is surprising given the fact that the very core of the dissonance process is the emotions evoked by dissonant behavior [5], [1]. In relation to this paucity in dissonance research, an unresolved issue has been to determine (a) to what extent (if any at all), the emotional reactions (both negative and positive) during the dissonance experience actually relate to the attitude-change effect (or other dissonance-reduction strategies), and (b) the valence of these relations. While no previous study has investigated this relationship in depth, there are some tentative (and mixed) results in the literature. For instance, Zanna and Cooper [6] found a positive relationship between the subjective experience of tension and attitude change. In another study, Pyszczynski et al. [7] found that when encouraging individuals (who experienced dissonance) to acknowledge their subjective feelings of anxiety and tension, they exhibited less attitude change. A more recent study using facial electromyography (fEMG) found that corrugator supercilii activation, an indicator of negative affect, was positively related to attitude change [8]. Other researchers have failed to find any relationship between emotional reactions and attitude change [9] [10], and have argued that there might not be a direct link between the emotional reaction to dissonance and dissonance-reduction strategies [11]. In the present study, we will attempt to clarify this issue by relating the dissonance process to appraisal theories of emotions, and emotion-regulation research.

### Dissonance reduction and emotions

A common thread in past dissonance studies examining emotions is that researchers have *not* asked individuals to appraise their emotions in relation to their dissonant behavior (e.g., writing a counter-attitudinal essay)–but rather their emotional state in general (“how do you feel right now?”). When asking individuals about emotions without relating them to a specific behavior, the researcher might actually collect data on current mood or general emotionality, rather than emotions produced by the dissonant behavior. Other researchers have focused on physiological reactions (e.g., electromyography or galvanic skin response) and taken these as evidence of negative emotions. However, appraisal theories of emotions hold that a specific emotional label depends on how the individual consciously appraises the situation [12]. For instance, the initial appraisal of the dissonant situation (agreeing to write a counter-attitudinal essay) might produce a physiological reaction in terms of negative arousal, which signals to the individual that something is out of the ordinary and should be attended to. However, the individual will not put a specific emotional label on the situation until a final cognitive evaluation has been made [13]. This means that two individuals could find themselves experiencing an equal amount of negative arousal in a given situation, but put completely different emotional labels to that same situation [14]. Thus, the conscious experience of emotions is a result of complex interactions between physiological reactions and cognitive appraisals [15], and measuring physiological reactions does not reveal how the individual mentally construes the aversive situation–which is the central part in the generation of emotions according to appraisal theories [15]. In the present research, we argue that in order to understand the relationship between dissonance reduction (e.g., attitude change) and emotions, one must examine the *consciously* experienced and expressed emotional label related to the *dissonant behavior* (i.e., writing the counter-attitudinal essay). Note that the individual might appraise an aversive situation in several different ways before making the final appraisal, especially in aversive situations that are novel [13]. Thus, the initial appraisal in an aversive situation is not necessarily how the individual will finally view the situation and feel about it. This kind of interplay between cognition and emotion is clearly depicted in the *process model of emotion regulation* (see Gross [16], for an overview). Specifically, the process model holds that individuals tend to engage in effortful cognitive work, called reappraisal, when it is not possible to avoid an aversive situation, modify it, or deploy attention elsewhere. Thus, in the reappraisal stage, the individual tries to see if the situation actually is as aversive as the initial appraisal suggests. The process model further suggests that the reappraisal of the aversive situation enables the individual to make a renewed global assessment of the situation. For instance, the initial appraisal of a dissonance situation (e.g., *I just agreed to write a counter-attitudinal essay*) might generate some negative arousal at first. However, since individuals can be assumed to try to reappraise the dissonant situation, this arousal does not automatically translate to experiencing full-blown negative emotions towards the situation. If the individual reappraises the situation by modifying cognitions to be consistent with the behavior (e.g., *maybe I do agree with the content of this essay*), it is even possible that the overall assessment of the situation will be positive (e.g., *now that I do agree with the content of the essay*, *I feel rather positive writing it*). A further reappraisal strategy to prevent the evolvement of negative emotions is to downplay the importance of the situation (e.g., *who cares about this anyway*), that is, to trivialize the dissonant behavior. The individual could also try to suppress the emotions altogether [16]. If the individual fails to change attitude, trivialize or suppress, either due to poor reappraisal abilities, or due to too vast discrepancy between the counter-attitudinal behavior and the initial attitude, the initial negative arousal could surely evolve into negative emotions. Hence, it is important to recognize that individuals will vary in how they resolve the dissonant situation. As a result of this variation, the valence and strength of emotions (assuming that emotions are directly related to the dissonance-reduction process), as well as their relations to attitude change, should also vary.

Based on these notions from emotion-regulation models, we expect that individuals who trivialize their behavior or suppress emotions will continue to mentally construe the situation as counter-attitudinal, although full-blown negative emotions might be prevented by these reduction strategies. Those similarly construing their behavior as counter-attitudinal but who do not trivialize the situation could be expected to be those most likely to experience negative emotions, but also those least likely to show attitude change. Since attitude change indicates that the individual now feels that the behavior is *pro*-attitudinal, it could further be expected that attitude change would be the dissonance-reduction strategy most clearly associated with positive emotions. This reasoning may seem counterintuitive given that *negative* emotions are the hallmark of attitude change in cognitive dissonance theory. But drawing on the process model of emotion regulation, and appraisal theories of emotions, it becomes clear that positive emotions might emerge in the midst of the dissonance-reduction process. Thus, in our view, dissonance theory could easily fit into the larger framework of general emotion regulation.

In sum, if the individual finds a way of integrating the dissonant behavior (writing the counter-attitudinal essay) and previous cognitions (initial attitude) into a coherent unity (attitude change), the initial negative arousal could in fact evolve into *positive* emotions when making the overall assessment of the situation. Those who trivialize the behavior or suppress their emotions could prevent the evolvement of negative emotions, but will not experience positive emotions towards the situation–since they will still view the behavior as counter-attitudinal. Finally, those who are unable to mentally or behaviorally solve the dissonant situation will likely experience negative emotions.

### General outline

In the current study, we will investigate the relationship between emotions and attitude change by asking participants to state how they felt with regard to both positive and negative emotions when *writing the counter-attitudinal essay*. In the two studies, we employ the widely used induced-compliance paradigm, in which students are asked to write a forceful counter-attitudinal essay. In this experimental procedure, individuals are typically given a perception of having low choice or high choice to engage in the action that is counter to an attitude they hold. Individuals who engage in the action with high choice have little justification for doing so (few consonant cognitions), and consequently, they typically experience dissonance [17–18]. In the current research, we only employ the high-choice condition, since we were interested in the emotional reaction when participants are experiencing dissonance. In the first study, we test our initial notion of a relation between emotions experienced *during* the counter-attitudinal behavior and attitude change. This is followed up by a second study to replicate the initial findings, controlling for baseline emotions and previous attitudes to the counter-attitudinal behavior that will be induced.

### Ethics

Both studies were conducted in accordance with the ethical principles outlined in European Code of conduct for Research Integrity Revised version [19]. The studies did not include factors that require ethical vetting according to Swedish legislation on research ethics, the act concerning the ethical review of research involving humans, SFS 2003:4, [20]. This was also confirmed by the head of the Department of Psychology at Stockholm University. In both studies, all participants were given the following information before giving consent: *All answers are anonymous and will only be analyzed statistically*, *which means that no individual's response can be traced in the presentation of this research*. *Note that you are entitled to cancel the study at any time without giving any explanation*. The first study was conducted online, and consent was obtained by participants clicking a box with the text *By clicking “Proceed to the study” you give your consent to participate in this study*. If this box was not checked, the participants could not continue to the experiment. In the second study, consent in the first step, information about the study and consent was given online as in study 1. In the second step (in the lab), participants were informed about the study and gave their consent orally. In the third step, which was conducted online, consent was obtained as in step 1.

### Hypotheses

Given our reasoning, we predict in both experiments that (a) positive emotions will be positively related to attitude change, and that (b) negative emotion experienced during the dissonance manipulation will be inversely related to attitude change. That is, the more negative emotions participants experience during the writing of the essay, the less attitude change, and the more positive emotions, the more attitude change.

### Study 1

In this experiment, we tested our hypotheses by conducting an online version of the induced compliance paradigm in which participants were asked to write a counter-attitudinal essay regarding animal testing in research.

## Materials and methods

### Participants

We recruited forty-eight undergraduate psychology students (mean age = 25.05, *SD* = 3.90, 75 percent women) who received course credits for their participation. Three participant were excluded for not following instructions (writing against the proposition instead of in favor), and one had already participated in a similar study. Thus, the final sample consisted of forty-four participants.

### Procedure and materials

Participants received an online survey where they were presented with a task that was introduced as a “creativity task”. This was done in order to mask the true purpose of the study. In the task, participants were asked to freely write an essay on the benefits of reducing some of the regulations regarding animal testing. They were informed that the government was planning on making a resolution that would make it easier to conduct animal research by reducing the amount of paper work, and controls. After writing the essay, participants were asked to indicate on a modified version of the PANAS [21] to what extent they experienced positive and negative emotions during the writing of the essay. This questionnaire had an 8-point scale (0 = Not at all; 7 = Very much) with the following emotions: *Joy*, *Discomfort*, *Optimism*, *Disappointment*, *Kindness*, *Guilt*, *Anxiety*, *Pride*, *Self-critique*, *Hope*, *Hostility*, *Excitement*, *Anger*, *Enthusiasm*, *Irritation*, *Inspiration*, *Shame*, and *Strength*. After stating their emotions, participants were asked to respond (on an 8-point scale) to some questions regarding the proposal: (1) *What is your opinion regarding the proposal on extended animal testing*? (0 = completely disagree; 7 = completely agree) (2) *What do you think other people’s opinion is on the proposal on extended animal testing*? (0 = completely disagree; 7 = completely agree) (3) *How difficult was it to write the essay*? (0 = not difficult at all; 7 = very difficult). The first question was the main dependent variable, and the other two questions were used as control questions. Studies show that young adults living in urban areas (e.g., university students) generally hold negative attitudes towards animal testing in research [22]. Our assumption in this study is that participants generally held a negative attitude towards the target issue when they entered the survey. Note, participants also completed additional personality questionnaires: Agreeableness and Openness from the Big Five [23], Need for Closure [24], and the Behavior Identification Form [25], however, these questionnaires were included for other purposes and will not be reported in this paper.

## Results and discussion

Because our hypotheses concerned the overall valence rather than the specific labels of the emotions, we made one index averaging the positive emotions (α = .93) and a second index averaging the negative emotions (α = .87), see Table 1 for means of positive and negative emotions and attitude after the dissonance induction. To investigate the relationship between the attitude towards the proposal (after writing the essay) and emotions, we ran a linear regression analysis using both positive and negative emotions as predictors, and the attitude as dependent variable. The regression model was significant *F*(2, 41) = 5.92, *p* = .006, *R*^*2*^ = .22, and further analysis revealed, as predicted, that positive emotions were positively related to participants’ attitude towards the proposal, *β* = .30 [CI 95% .03, .58], *t*(2, 41) = 2.21, *p* = .03, whereas negative emotions were inversely related to their attitude, *β* = -.38 [CI 95% -.66, -.10], *t*(2, 41) = 2.73, *p* = .01. Note that task difficulty was not related to attitude towards animal testing (*β* = .19, *p* = .249) when included in the regression model together with positive and negative emotions. And it did not alter the relationship between emotions and attitude either, as positive and negative emotions remained significant. When entering the belief about others’ attitude towards animal testing in the regression model (excluding task difficulty, but keeping positive and negative emotions as predictors), the results revealed that belief about others’ attitude was positively related to one’s own attitude (*β* = .32, p = .03). However, again, this did not alter the relationship between emotions and attitude, as all predictors were significant in this model. In sum, results reveal a clear relationship between emotions (positive and negative) and attitude, and this relationship was not influenced by task difficulty, or belief about others attitude.

**Table 1:** Mean and (standard deviation) for attitude after dissonance induction, and emotions associated with the same dissonance induction.

	Mean (SD)
**Attitude**	1.27 (1.84)
**Positive emotions**	1.15 (1.28)
**Negative emotions**	2.87 (1.54)

Attitude scale ranged from 0 (Completely disagree) to 7(Completely agree). Emotion scale ranged from 0 (Not at all) to 7 (Very much).

Our first experiment thus sends an encouraging message with respect to our hypotheses. In line with our predictions, positive emotions seem to be associated with a more pro-animal testing attitude after writing the essay, whereas negative emotions are inversely associated with this attitude. Thus, asking participants to relate their emotions to the specific attitude-discrepant behavior (writing the essay), instead of assessing their emotions in general, suggests an emotional regulation process, more complex and dynamic than previously hypothesized or demonstrated, but well aligned with emotion regulation and appraisal theories.

However, this first experiment suffers from shortcomings which prevent strong conclusions. First, while we assumed, based on studies on attitudes toward animal testing in research [22], that the majority of participants had a negative attitude towards the target topic before writing the essay, we did not actually measure their pre-experimental attitude. Hence, whether there was an actual change in attitudes is not clear. Moreover, as we did not measure participants’ baseline emotions, such basic levels may constitute a potential confound. In the next experiment, we aimed at replicating the results with a study conducted in the lab and with a different version of the induced compliance paradigm. In this experiment, we measured participants’ attitudes towards the target issue and emotions on three different occasions; (1) several days before the dissonance induction, (2) directly after the dissonance induction, and (3) several days after the dissonance induction. With these three measures across time points, we can establish whether we are relating emotions to actual attitude *change*, rather than just the expression of a momentary attitude, and control for participants’ baseline emotions before and after the intervention. Moreover, we can examine whether the attitude change holds for an extended period after the expected initial attitude change.

### Study 2

To further test our hypotheses, we conducted a second study with a different version of the induced compliance paradigm. We created a fictitious proposition suggesting that students should lose 20 percent of their student aid for every exam they failed. In Sweden, universities are tuition-free, but most students are dependent on financial aid from the government. Students receive student aid of approximately $1233 a month. About two-thirds of this amount ($826) is a government loan (with substantially lower interest than commercial banks). The remaining one-third ($407) is a grant administrated by the National Board of Student Aid (Centrala Studiestödsnämnden). This particular version of the induced compliance paradigm has been used, successfully, in a previous study (see Cancino-Montecinos et al., [18]). Several days before, and several days after the actual experiment, we measured participants’ attitudes and their baseline positive and negative emotions.

## Materials and methods

### Participants

We recruited 127 Stockholm university students (mean age = 25.47 years, *SD* = 6.39, 65 percent women), who received movie vouchers for their participation. Eleven participants who indicated a positive attitude toward the proposition in the pretest (a score of 4 or higher on an 8-point scale) were excluded, and an additional eight participants who failed to follow instructions (e.g., writing strongly against the proposition) were excluded. Finally, one participant was excluded for not completing the final part of the experiment, and another for having participated in a similar study before. In total, twenty-one participants were excluded, leaving 106 participants for the analysis.

### Procedure and materials

#### Step 1 –Before dissonance induction

Before the actual dissonance manipulation participants received an online survey that began with a modified version of the PANAS [21], to establish their baseline emotions in a neutral situation. In the PANAS participants had to indicate on an 8-point Likert scale (0 = Not at all; 7 = Very much) to what extent they experienced both positive and negative emotions *at the moment*. This questionnaire consisted of the following emotions: *Joy*, *Discomfort*, *Optimism*, *Frustration*, *Kindness*, *Guilt*, *Anxiety*, *Pride*, *Fear*, *Hope*, *Hostility*, *Excitement*, *Anger*, *Enthusiasm*, *Relief*, *Irritation*, *Inspiration*, *Shame*, *Nervousness*, *Strength*, and *Sadness*. As in Experiment 1, we averaged the positive emotions to create an index (α = .90) and a second index consisting of negative emotions (α = .89). After the PANAS, participants were asked to state their opinion (on an 8-point scale, 0 = Completely disagree; 7 = Completely agree) on five different statements regarding their university. After each statement, they were also asked to indicate how important the previous question was to them (on an 8-point scale, 0 = Not important at all; 7 = Extremely important). The target statement was: *students should lose 20 percent of their student aid for each failed exam*, *meaning that students’ monthly aid of $1233 should be reduced to $986 the same month they fail the exam*. After completing the online survey, participants were scheduled for the second part of the study. The mean time elapsing from first and the second part was 9.15 (*SD* = 5.80) days. Note, participants were also asked to complete additional questionnaires in step 1: Adult Temperament Questionnaire [26], Self-Monitoring Scale [27], Self-Consciousness Scale [28], Behavior Identification Form [25], Preference for Consistency [29], Trait-Meta-Mood Scale [30], Emotion Regulation Questionnaire [31]. These questionnaires were part of a subproject involving a comparison between a specific version of the free-choice paradigm and the induced compliance paradigm in terms of temperament and memory distortions. The aim of that subproject is to investigate whether temperamental reactivity predicts people’s recollection of past choices. More precisely, the aim was to study whether this reactivity is related to people’s tendency to remember past choices as more favorable. We also wanted to investigate the pattern of correlations between the temperament measures and the other measures in order to understand the commonalities between temperament and other relevant psychological constructs. Finally, we were also interested in the psychometric properties of all the measures, and thereby maybe exclude those that do not meet the common criteria found in the psychometric literature. However, all these aims were for the above-mentioned subproject. Thus, these measures will not be reported in this paper.

#### Step 2 –Dissonance induction

In this part of the study, participants arrived at the lab presumably to perform a series of cognitive tests. However, the experimental session began with the experimenter asking participants if they wanted to volunteer to participate in a survey administered by the university. The question was asked as follows:

While I get the computer ready, would you like to participate in a survey administered by the university? The university is trying to map students’ opinions regarding different issues and they would be very grateful if you could participate. After finishing this, we will proceed with the cognitive tests.

Information about the university survey (and a sheet of paper to write on) was placed in an envelope that participants received when agreeing to participate. All agreed to write the essay. The instructions in the envelope read: *because we already have enough participants arguing against the proposition*, *we would like for you to freely write an essay in favor of the proposition*. After writing the essay, participants received the same modified version of the PANAS as in part one, but this time they were asked to indicate to what extent they had experienced positive (α = .91) and negative emotions (α = .88) *while writing the essay*. After stating their emotions, participants were asked to answer some “control questions” regarding the proposition: (1) *How voluntary did you experienced the task to be*? (0 = Not voluntary at all; 7 = Completely voluntary), (2) *What is your opinion regarding the student aid proposal*? 0 = Completely disagree; 7 = Completely agree), (3) *How important is the student aid proposal for you personally*? (0 = Not important at all; 7 = Extremely important), (4) *How engaged were you in writing the essay*? (0 = Not engaged at all; 7 = Extremely engaged), (5) *Are you currently receiving student aid*, *Yes or No*? After completing these questions, participants performed cognitive tests; which were administered to mask the true purpose of the experiment. The tests consisted of the Gestalt Completion Test (GCT) [32], and a shorter version of the Wisconsin Card Sorting Task (WCST) [33]. In the GCT, participants’ task is to identify specific objects in drawings that are composed of black blotches representing only parts of the object being portrayed. In the WCST, participants need to identify what rule to apply when trying to match a given card with four other cards. The rule can either apply to the color, shape, or the number of symbols found on the given cards. After completing the cognitive tests, participants were informed that they would receive the third and final online survey in a few days.

#### Step 3 –After dissonance induction

In the third and final part of the study, on average, 6.44 (*SD* = 4.39) days after the second part, participants received an online survey where they were asked to complete some control questions regarding previous parts of the study. The survey began with the modified PANAS that aimed at measuring participants’ baseline emotions for the second time. Since we aimed to investigate if attitude change from part 1–3 was influenced by participants’ baseline emotions expressed at the same time as expressing attitude for the third time, we measured baseline emotions in conjunction with the third measure of attitude. As in part 1, participants were asked to state how they felt at the moment, and then we made indexes of positive (α = .92) and negative emotions (α = .90). Afterwards, they were asked the control questions in the following order: (1) *How did you experience the questionnaires from part 1 of the study*? (0 = Not at all difficult to answer; 7 = Extremely difficult to answer); (2) *In the second part*, *you had to write a text about the reduction of student aid in case of failed exams*. *What do you think of that proposal*? (0 = Completely disagree; 7 = Completely agree); (3) *How important is the proposal about student aid reduction for you personally*? (0 = Not important at all; 7 = Extremely important); (4) *How did you experience the task in which you had to figure out what the blotches of ink represented*? (0 = Not at all difficult; 7 = Extremely difficult); (5) *How did you experience the task in which you had to figure out what rule (color*, *form or number of symbols) to follow*? (0 = Not at all difficult; 7 = Extremely difficult); (6) *What do you believe was the hypothesis of the study*? *That is*, *what do you think the true purpose of the study was*? The key questions in this survey were number 2, 3 and 6, and the PANAS. Question 2 was asked in order to investigate if participants changed their opinion regarding reduction of student aid, or if they stuck to their opinion from part 2 and part 1. The third question was asked for the same reason as the second question, that is, if they changed their mind on the matter. In this case, it concerned how important they felt the student aid proposal was to them personally. The final question was to make sure that participants had not figured out the true purpose of the study–which no one did. The other questions (1, 4 and 5) were used as fillers.

## Results and discussion

### Attitude change

In this study, we measured participants’ attitudes to the proposition on three different occasions. Our main dependent measure was the attitude change that occurred from the first (before the dissonance induction) to the second part (immediately after dissonance induction) of the study. A paired samples t-test revealed that overall, participants significantly changed their attitude after the dissonance manipulation, *t*(1, 105) = 5.30, *p* < .001, *d* = .55. More specifically, participants were generally more positive to the proposal after writing the essay than they were initially. A second paired samples t-test was run to investigate if the attitude-change effect remained several days after the dissonance manipulation. Results revealed that the attitude-change effect was significant in the third part (vs. part 1) of the experiment as well, *t*(1, 105) = 4.49, *p* < .001, *d* = .50. A third t-test revealed that there was no difference in participants’ attitude towards the proposition from step 2 (immediately after dissonance induction) and step 3 (1 week after dissonance induction), *t*(1, 105) = .86, *p* = .39. This final analysis suggests that the attitude-change effect is rather stable over time (see Table 2).

**Table 2:** Mean and (standard deviation) for attitude towards the proposal and emotions across step 1–3 in Experiment 2.

	Step 1 1 week before dissonance induction	Step 2 Immediately after dissonance induction	Step 3 1 week after the dissonance induction
**Attitude**	.58 (.92)	1.33 (1.74)	1.25 (1.70)
**Positive emotions**	3.25 (1.31)	1.63 (1.32)	2.81 (1.28)
**Negative emotions**	1.58 (1.16)	2.08 (1.34)	1.22 (1.04)

Attitude scale ranged from 0 (Completely disagree) to 7(Completely agree).

Emotion scale ranged from 0 (Not at all) to 7 (Very much).

In sum, these results reveal an overall stable (over time) medium-sized attitude-change effect. However, a closer look at the data reveals that attitude change is not used by all participants as a way of reducing dissonance, but the attitude-change effect is quite large for those who actually do change their attitudes. Descriptive statistics show that 38.7 percent (41 participants) changed their attitude towards being more positive regarding the target issue compared to their initial standpoint, 55.7 percent (59 participants) did not change their attitude, and 5.7 percent (6 participants) became more negative towards the target issue after the manipulation. Participants who changed their attitude did so on average 2.20 steps (*SD* = 1.31) on the eight-point scale (0–7), *d* = 1.62. Thus, as mentioned in the introduction, people vary in their use of attitude change as a dissonance-reduction strategy. In the next section, we will test if this attitude-change effect is related to emotions.

### Attitude change and emotions

We had hypothesized that, as in Experiment 1, negative emotions during the writing of the essay would predict less attitude change, and positive emotions would predict more attitude change, and that these effect would hold even when controlling for baseline emotions. A hierarchical linear regression analysis was conducted, with attitude change (1 week before the dissonance induction minus attitude immediately after dissonance induction) as outcome variable, and baseline positive and negative emotions 1 week before dissonance induction as predictors in model 1, and positive and negative emotions associated with writing the essay (immediately after dissonance induction) as predictors in model 2. The first model, with baseline positive and negative emotions as predictors, was not significant, *F*(2, 103) = .36, *p =* .70. The second model, adding positive and negative emotions experienced during the writing of the essay as predictors, yielded a significant change relative to model 1, *F*(4, 101) = 18.55, *p* < .001, Δ*R*^*2*^ = 27. Specifically, the analysis revealed that negative emotions were inversely related to attitude change, whereas positive emotions were positively related to attitude change (see Table 3). Baseline emotions remained non-significant in the second model.

**Table 3:** Beta weights for the relationship between positive and negative emotions from step 2 and attitude change from step 1–2 and 1–3, controlling for baseline emotions from step 1 and 3.

Regression with attitude change from step 1–2 as outcome variable	*β* (CI 95%)	Regression with attitude change from step 1–3 as outcome variable	*β* (CI 95%)
**Model 1**		**Model 1**	
Positive emotions step 1	.07 (-.12, .27)	Positive emotions step 1	.11 (-.08, .31)
Negative emotions step 1	.05 (-.15, .25)	Negative emotions step 1	.12 (-.08, .31)
**Model 2**		**Model 2**	
Positive emotions step 1	-.02 (-.20, .16)	Positive emotions step 1	.03 (-.16, .22)
Negative emotions step 1	.01 (-.18, .19)	Negative emotions step 1	.07 (-.13, .26)
Positive emotions step 2	.45 (.27, .64)**	Positive emotions step 2	.39 (.20, .58)**
Negative emotions step 2	-.22 (-40, -.04)*	Negative emotions step 2	-.15 (-.34, .04)
		**Model 3**	
		Positive emotions step 1	-.03 (-.25, .19)
		Negative emotions step 1	.02 (-.21, .26)
		Positive emotions step 2	.36 (.16, .56)**
		Negative emotions step 2	-.16 (-.36, .03)
		Positive emotions step 3	.11 (-.11, .33)
		Negative emotions step 3	.06. (-17, .29)

* *p* < .05

** *p* < .001

To examine if the relationship between attitude change and emotions experienced during the dissonance induction held with the attitude as measured 1 week after the dissonance induction, a second hierarchical linear regression analysis was run using attitudes from step 3 (1 week after dissonance induction) minus attitudes from step 1 (1 week before dissonance induction) as outcome variable, and baseline emotions from step 1 (1 week before dissonance induction) as predictors in the first model, emotions from step 2 (dissonance induction) in the second model, and baseline emotions from step 3 (1 week after dissonance induction) in the third model. The first model, with baseline emotions from step 1 as predictors, was not significant, *F*(2, 103) = 1.20, *p =* .31, *R*^*2*^ = 02. The second model, adding positive and negative emotions from step 2 as predictors, was significant relative to the first model, *F*(2, 101) = 11.11, *p* < .001, Δ*R*^*2*^ = 18. The third model, adding baseline emotions from step 3, was not significant relative to the second model *F*(2, 99) = .58, *p* = .56, Δ*R*^*2*^ = 01. Thus, attitude change from step 1 to step 3 is associated with emotions experienced during the dissonance induction, not baseline emotions. This time however, about a week after the dissonance manipulation, only positive emotions experienced during the writing of the essay were a clear significant predictor of attitude change (see Table 3). However, the pattern of the relationship between negative emotions (experienced during the writing of the essay) and attitude change was in the predicted direction.

Replicating the findings from our first study, the results of Experiment 2 clearly revealed that negative and positive emotions experienced during the dissonance manipulation do explain variance in attitude change. This relationship holds also when controlling for baseline positive and negative emotions. As opposed to study 1, however, these results do in fact confirm that emotions were related to attitude *change*. That is, in study 1 we could not confirm that emotions were related to attitude change, since we did not have a pre-measure of participants’ attitude. Thus, we could not exclude that participants experienced more positive emotions towards the proposal because they already had a positive attitude towards the proposal. In this study, we can exclude this alternative explanation. Overall, our two experiments attest to the value of approaching the mechanisms behind dissonance reduction from the larger framework of general emotion-regulation processes, and appraisal theories of emotions.

## General discussion

The concept of cognitive appraisals, and emotion regulation, may seemingly lie outside the realm of cognitive dissonance theory. In this study, however, we have argued that these concepts belong to the very core of it. We found (in two studies) that negative emotions experienced during cognitive dissonance were in fact inversely related to attitude change, whereas positive emotions experienced in the same dissonance situation were positively related to attitude change. This relationship remains several days after the dissonance manipulation–especially for positive emotions. Thus, asking participants about their emotions concerning their dissonant behavior, rather than asking about their general emotional state, or measuring their physiological reactions, does reveal important links between emotions and attitude change.

Confirming the relevance of appraisal theories and emotion regulation research to cognitive dissonance, the findings of the current study are consistent with the idea that the dissonance process might be a rather dynamic process in which people’s initial negative arousal does not necessarily translate to negative emotions, and where cognitive reappraisals might play a vital role in the relationship between emotions and attitude change. Thus, compared to the original (linear) conceptualization of the dissonance process (**A** = inconsistent cognitions → **B** = dissonance arousal → **C** = reduction strategy implemented → **D** = dissonance alleviation [34] [1], we argue for a more complex process where cognitive appraisals go back and forth until the individual finds a stable (and coherent) understanding of the situation, see Scherer [13]. For example, in the present study, one can speculate that participants presumably felt some negative arousal when faced with the reality of writing the essay–since the proposal was rather important to them, and they disagreed with it beforehand. However, once the writing begins, some participants might convince themselves (by reappraising the current behavior in relation to previous attitudes) that there are actually some benefits with the proposal. Others will write the essay but might not change their attitude. In this group of participants, there are perhaps some that are good at regulating, and downplaying their negative emotions (without changing attitude), while others might continue to feel negative emotions. Thus, when asked to what extent they experienced both negative and positive emotions during the writing of the essay, participants who change their attitude might perceive this episode as positive since they now are more in favor of the proposal. Future research could explore this reasoning more closely, for example by employing a think-aloud protocol during the essay writing. Such a method would allow following participants thought processes in a more detailed way, and clarify mechanisms during the dissonance-reduction process. To our knowledge, no previous study has employed a think-aloud protocol in the induced compliance paradigm.

Based on the reasoning above, we suggest that dissonance theory might fit in the larger framework of general emotion-regulation processes. Thus, the dissonance-reduction process could be conceptualized as a special case of emotion regulation. This special case might then be characterized by the extensive reappraisal effort executed by the individual—which is possibly the driving force in the attitude-change effect. Note that we argue that trivialization might also come from reappraisal, however, trivialization involves changing the meaning of the situation, rather than changing attitudes so they fit with behavior. An interesting avenue for future research should therefore be to disentangle the difference, in terms of reappraisal, between these two types of dissonance-reduction strategies. Interestingly, research findings suggest that there indeed are different types of reappraisals [35]. For instance, the individual could reappraise of the *emotional stimulus* (e.g., the dissonance situation) and/or reappraise the *emotional reaction* (e.g., the initial negative arousal). A third way of reappraising a situation is *perspective taking*, which involves assessing the situation in a more objective manner by trying to detach oneself from it. Research also shows that people sometimes use a mixture of different types of reappraisals [35]. Thus, a plausible assumption is that the difference between attitude change and trivialization lies in different mixtures of reappraisals. Note however that individuals could also change attitude *and* trivialize, making the differentiation difficult to identify. Furthermore, suppression can also be done in different ways. For instance, the individual could try to suppress the *expression* of the emotions, and/or try to suppress the *experience* of the emotions. A third way is trying to suppress the thoughts of the emotion-eliciting event. Thus, the regulation of the initial negative arousal, and the prevention of negative emotional evolvement, could be a result of different processes (reappraisal or suppression) and/or a mixture within these processes. This shows that the dissonance-reduction process might be more complex than earlier conceptualizations of the dissonance theory thought it to be [36–37], [1], [11]. One way for future research to disentangle the difference between dissonance-reduction strategies could be to investigate what individual differences correspond uniquely with attitude change and trivialization. To date, the study of individual differences in dissonance reduction is rather scarce, scattered and ambiguous (see e.g., Harmon-Jones et al., [11]), and has focus almost exclusively on attitude change. Furthermore, these studies have focused more on personality-related differences (e.g., self-esteem [38], preference for consistency [29], behavioral approach [39]). However, if the dissonance-reduction process truly is a part of the larger framework of general emotion regulation, then people’s differences in their habitual use of emotion-regulation strategies (or coping strategies) could reveal some important insights, and would make a significant contribution to dissonance research.

Although the current findings show that negative *and* positive emotions are related to attitude change, they do not reveal whether positive emotions and attitude change are activated simultaneously or sequentially. Furthermore, the exact mechanisms behind different dissonance-reduction strategies (e.g., attitude change vs. trivialization) need to be further explored. One should also further explore to what extent positive emotions (experienced during the dissonant situation) is a reflection of people simply being satisfied with their essay, as opposed to them having changed attitude. It could also be the case that people feel more satisfied with their essay if they manage to change attitude, and the following positive emotions might be a reflection of a mixture of satisfaction *and* attitude change. Finally, the findings relate specifically to the induced compliance paradigm, future research should therefore try to investigate if these results hold in other dissonance-research paradigms–for instance the free-choice paradigm [40].

## Conclusions

The present study resolved an old dissonance-research conundrum of how emotions relate to the classic attitude-change effect. These findings have important implications for the understanding of the regulatory process involved in cognitive conflicts. Future research should try to shed more light on the details of this process, and relate it to other relevant theories.
